# A Cluster Randomized-Controlled Trial of a Classroom-Based Drama Workshop Program to Improve Mental Health Outcomes among Immigrant and Refugee Youth in Special Classes

**DOI:** 10.1371/journal.pone.0104704

**Published:** 2014-08-15

**Authors:** Cécile Rousseau, Caroline Beauregard, Katherine Daignault, Harriet Petrakos, Brett D. Thombs, Russell Steele, Helen-Maria Vasiliadis, Lily Hechtman

**Affiliations:** 1 Division of Social and Cultural Psychiatry, McGill University, Montreal, Quebec, Canada; 2 Department of Educational Psychology and Adult Education, University of Montreal, Montreal, Quebec, Canada; 3 Department of Mathematics and Statistics, McGill University, Montreal, Quebec, Canada; 4 Department of Education, Concordia University, Montreal, Quebec, Canada; 5 Department of Psychiatry -Lady Davis Institute for Medical Research, McGill University, Montreal, Quebec, Canada; 6 Department of Community Health Sciences, Sherbrooke University, Sherbrooke, Quebec, Canada; 7 Division of Child Psychiatry, McGill University, Montreal, Quebec, Canada; University of Western Brittany, France

## Abstract

**Objectives:**

The aim of this cluster randomized trial was to evaluate the effectiveness of a school-based theatre intervention program for immigrant and refugee youth in special classes for improving mental health and academic outcomes. The primary hypothesis was that students in the theatre intervention group would report a greater reduction in impairment from symptoms compared to students in the control and tutoring groups.

**Methods:**

Special classrooms in five multiethnic high schools were randomly assigned to theater intervention (n = 10), tutoring (n = 10) or control status (n = 9), for a total of 477 participants. Students and teachers were non-blinded to group assignment. The primary outcome was impairment from emotional and behavioural symptoms assessed by the Impact Supplement of the Strengths and Difficulties Questionnaire (SDQ) completed by the adolescents. The secondary outcomes were the SDQ global scores (teacher and youth reports), impairment assessed by teachers and school performance. The effect of the interventions was assessed through linear mixed effect models which incorporate the correlation between students in the same class, due to the nature of the randomization of the interventions by classroom.

**Results:**

The theatre intervention was not associated with a greater reduction in self-reported impairment and symptoms in youth placed in special class because of learning, emotional and behavioural difficulties than a tutoring intervention or a non-active control group. The estimates of the different models show a non-significant decrease in both self-reported and impairment scores in the theatre intervention group for the overall group, but the impairment score decreased significantly for first generation adolescents while it increased for second generation adolescents.

**Conclusion:**

The difference between the population of immigrant and refugee youth newcomers studied previously and the sample of this trial may explain some of the differences in the observed impact of the theatre intervention.

**Trial Registration:**

ClinicalTrials.gov NCT01426451

## Introduction

Schools have a central role in mental health promotion and problem prevention. Within schools, children and adolescents identified as having emotional and behaviour disturbances (EBD) represent a challenge. Two meta-analyses have evaluated the effectiveness of school-based interventions in self-contained special education classrooms with this population. Reddy and al. [1] reviewed 29 trials and found that, overall, school-based programs significantly reduced emotional symptoms and sometimes increased academic performance. Wilson and Lipsey [2] focused on aggressive and disruptive behaviours and also found that overall, school-based programs decreased these behaviours in the 249 trials they reviewed. However, the magnitude of effects were small (mean effect size of 0.20 to 0.35), and effectiveness depended on the particular school settings or populations where interventions were delivered.

In the field of ethnic studies, the ethnic and racial biases underlying referral to special classes have been studied extensively in the United States over the last decades, and recent studies have also highlighted the adverse effects of segregation from peers having weak academic performance for minority group children, who are over represented in disadvantaged schools [3]–[5]. Immigrant parents fear stigma and often perceive specialized services and classes as a threat [6], [7]. Given the views that their families and communities associate with being assigned to a special class, and given the social position attached to minority cultural status in economically socio-disadvantaged neighbourhoods, these youth often experience a double exclusion [8]. Few intervention programs, however, have addressed the specificity of this group of youth: being of immigrant origin, identified as having emotional, behavioural and / or learning difficulties and being assigned to a special class.

The field of immigrant and refugee studies emphasises the role of schools to implement prevention programs based on ecological principles that enhance young people's ability to adapt to life circumstances [9]–[11]. Psychotherapy (mostly cognitive behavioural therapy), creative play, and mixed modalities have been shown to be effective to improve mental health for immigrant and refugee children [12] or for children which, like many of them, have experienced an armed conflict [13]–[15]. Building on the literature describing the usefulness of creative expression programs for immigrant and refugee children in clinical and community settings [16]–[20], a Montreal Team composed of schools, community organizations and health professionals developed a set of creative expression arts-based prevention programs for preschools, elementary schools and high schools. The aims of these programs are to help immigrant children and adolescents bridge the gap between home and school and past and present and to work through experiences of loss and trauma. In high schools, qualitative evaluation of drama workshops in classes for newly arrived migrants suggests that they provide a safe expression space in which adolescents benefit from the support of their peers, the team and the ritual nature of the activity. The drama workshops promote the development and assimilation of the different transitional experiences associated with adolescence, migration and having a hybrid identity and enable young people to transform, at least in part, their experiences of adversity into a learning opportunity [21]. In a previous study of the theatre workshop program with adolescents in classes designed to provide immigrant children in Quebec, Canada with French language instruction prior to integration into mainstream classes (n = 123) [22], a significant reduction (p<0.05) in the perception of distress and impairment associated with mental health symptoms was reported by adolescents of the experimental group compared with those in the control group (*β* = −0.194). With respect to academic performance, results indicated a significant improvement (p<0.05) in performance in math among the adolescents who took part in the activity compared with those in the control group (*β* = −0.167). The teachers in the experimental groups also reported an increase in the general participation level of their students in class compared with the teachers in the control group.

In a subsequent pilot study of the drama workshops in classes of under schooled youth (with at least 4 years of academic delay), the impairment significantly decreased in the experimental group (n = 27) (paired T test  = 2,443, p: 0.021) but not in the control group (n = 28) [23].

The workshops were subsequently adapted for special classes of immigrant and refugee adolescents with emotional, behavioural or learning difficulties. The workshops were designed to transform teachers' perceptions of the adolescents by highlighting not only student experiences of adversity (trauma, grievance, discrimination), of which the teachers were often unaware, but also their resources and strengths, which are often not recognized in the usual school context [24].

The present project builds on this previous work in two ways: (1) This is the first randomized trial of the use of theatre as a means of helping immigrant youth in special classes due to learning and behavioural problems; (2) The past study did not include an active control intervention to control for the increased attention received by the youth during the intervention. This is important because recent school-based RCTs of CBT interventions have shown that non-specific factors, and in particular positive reinforcement, may play a key role in the students' improvement [25].

The aim of this study was to evaluate the effectiveness of a school-based theatre intervention program for immigrant and refugee youth in special classes for improving mental health and academic outcomes. The primary hypothesis was that students in the theatre intervention group would report a greater reduction in impairment from symptoms (emotional and behavioural) compared to students in the control and tutoring groups from pre-intervention (T0) to the end of the 12-week intervention program (T1).

Secondary hypotheses were that compared to students in the tutoring and control groups, students who received the theatre program intervention would report a greater decrease in global emotional and behavioural symptoms per self- and teacher-report from T0 to T1 and that compared to students in the control groups, students who received the theatre program or the tutoring intervention would report a greater improvement in school performance in their main subjects (French and Math) compared to the control group.

## Methods

The protocol for this trial and supporting CONSORT checklist are available as supporting information; see Checklist S1 and Protocol S1.

Ethics approval was obtained from the Ethic Review Board of the CSSS de la Montagne. Consent was obtained from the parents (or guardian) and assent was obtained from the adolescents. Both consent and assent were written on forms which had been approved by the Ethics Review Board.

### 1. Setting and participants

**Figure 1:**
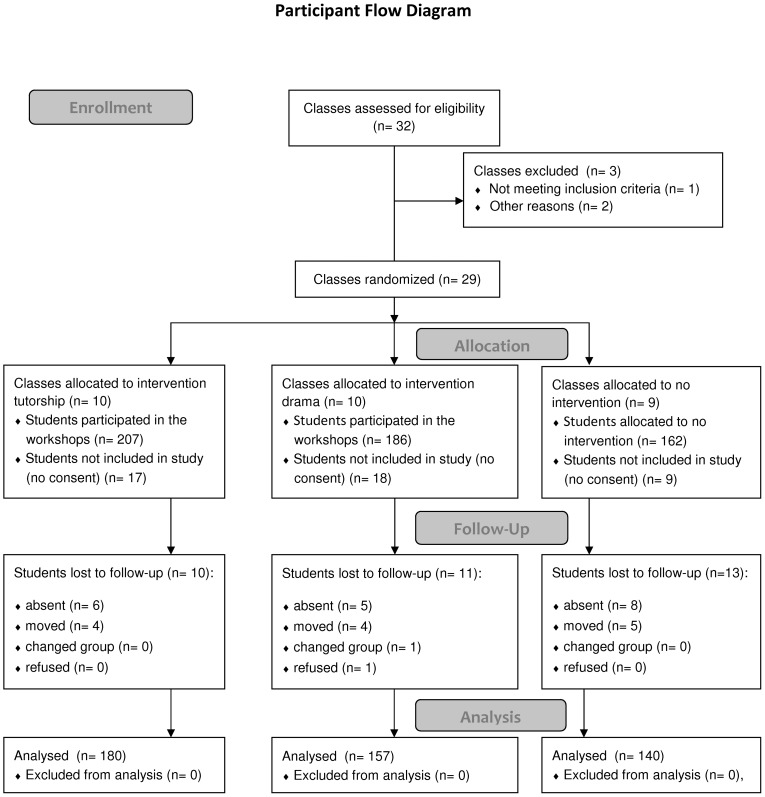
Participant Flow Diagram.

The target population for the intervention was made up of secondary school students attending a high multiethnic density school who were assigned to special classes, because of learning or behavioural problems. These classes are comprised of youth who typically have at least 2 years of academic delay. One third of the classes are composed of youth who have not finished their elementary school curriculum. A second third is composed of 7^th^ graders, while the third group consists of 8^th^ graders. The five schools taking part in the program and its evaluation were selected on the basis of their interest in these projects and because they serve disadvantaged, highly multiethnic communities and have a large number of special classes. Students in ten special classes received the theatre program, students in ten other classes received the group tutoring activity, and students in nine others constituted the control group. The sample size was determined based on an estimate of 18 students per class, a compliance rate of 80% and a 5% loss to follow-up at T1. Within-class variance and residual variance were estimated based on data from a pilot project. However, the pilot project was carried out with newly arrived immigrants, whose symptoms generally tend to decrease rapidly with time [26] while in the present study, participants were mostly first and second generation immigrants who have been presenting emotional and behavioural problems chronically. Thus, power calculations took that information into account and the sample size had a 77.5% power to detect a moderate effect size (d = 0.50) corresponding to a 3.95 change in the SDQ impairment score. Students who declined to participate in the research nevertheless attended the workshops that were part of the regular class curriculum, but they did not participate in the data collection. The ideal evaluative research design would have required that all students who agree to take part in the research project be distributed randomly among the experimental and control groups. Constraints imposed by the functioning of the secondary schools did not allow this, and the evaluative strategy is therefore based on school stratified random assignment to experimental or control status. In an initial stage, a list of the special class homeroom teachers was drawn up to form 5 sampling pools, one for each high school. In a second stage, 2 experimental classes, 2 tutoring classes and 2 curriculum as usual classes were randomly chosen by a research assistant from each of the 5 sampling pools/schools for a total of 29 classes (Figure 1). Students and teachers were non-blinded to assignment.

All students who were assigned to special classes based on behavioural or learning problems in grades 7–10 in the participating schools were eligible for the study. We decided not to exclude the more severe cases, although it might have facilitated the implementation of the intervention [13], [27] because it would not have represented the everyday challenges that teachers encounter in special classes, and because real classroom settings provide a better measure of effectiveness [28].

At the start of the school year, the schools sent study participation consent forms to the parents and assent forms to students. Recruitment took place from November 2011 to December 2011 (T0). T1 data was obtained from May 2012 to June 2012. Overall 88.8% of students agreed to participate and received parental permission (Figure 1). Loss to follow up was 6.7% (n = 34) and was attributed to absences (3.7%; n = 19) moving out (2.5%; n = 13), change of group (0.2%; n = 1) and refusal (0.2%; n = 1).

### 2. Intervention

#### Theatre expression workshops

The purpose of the theatre expression program adapted to special classes for students with learning and behavioural problems is to facilitate the expression and sharing of group stories by young immigrants and refugees in order to (1) give support to the construction of meaning in an individual's personal history; (2) foster the grieving processes associated with immigration (separation, transition, loss of expectations) and experiences of academic failure; (3) help consolidate multiple identities; (4) enhance appreciation of differences and the development of adaptation strategies without increasing marginalization and exclusion. These processes are expected to help alleviate problems associated with distress and behaviour stemming from the losses of migration and the tensions of belonging to a minority group in the host society, and of being identified as being problems by the school. They can also foster social adjustment and academic performance of adolescents.

The theatre expression program is a manualized intervention, running for 12 weeks, with one 90-minute workshop per week. The workshops are incorporated into the regular class timetable and are run by two members of the intervention team who have training in theatre and psychology, with the support of the teacher, whose level of direct involvement increases gradually as he or she becomes familiar with the workshops. The workshop leaders act as guides and encourage the participants to improvise verbally, musically and through gestures, and to explore alternative scenarios. The workshops are structured on the basis of two types of activities:

Warm-up: generally for the first two meetings (sessions 1 and 2), exercises and games focusing on listening, trust and non-verbal expression help participants get to know each other while encouraging play, humour and imagination. The warm-up exercises are done in a respectful, tolerant environment that allows for individual differences while building group solidarity.

Improvisation: Theatrical methods (e.g. fluid sculptures and ambivalent situations), inspired from youth personal stories related to a weekly theme, are used to familiarize the participants with improvisation. Later, full stories contributed by the adolescents are explored, played out and validated. When an expression of distress demonstrates a lack of emotional regulation or illustrates a situation of exclusion or discrimination (Boal transformation), alternative scenarios are developed collectively and contribute to personal and group empowerment. Over the course of the 12 workshops, a variety of topics are explored. In the program adapted to the needs of special classes, the themes are: belonging and exclusion, learning (especially experiential learning), family and friends (evoking issues of solidarity and social network) and the topic of transitions (migration, adolescence or other turning points in the youth's experience).

#### Group tutoring program

The theatre workshops are compared with a program in the context of a self-contained classroom. Irrespective of the classroom placement (special or general education classes), the interventions for students with EBD almost exclusively focus on behavioural management with academic learning being addressed secondarily, if at all [29]. These students are often achieving significantly below level as compared to students without disabilities and exhibit underachievement in all academic areas [30]. Research also consistently indicates that they do not improve over time and that in some areas (e.g., math) their difficulties increase (see review [31]). In addition, studies on high school students with EBD in a self-contained setting indicated that overall school adjustment was predictive of academic performance. Therefore, the comparison intervention of the present study was an academic in-class intervention that focused on differentiated academic instruction and on improving overall academic adjustment. Each self-contained class includes a core teacher who teaches curricula based on the Quebec Education Program with grade appropriate competencies in reading, math, social studies and science content areas. In each classroom assigned to the tutorship intervention, two academic resource assistants provided weekly in-class support to students for the same length of time as the drama workshops. Individualized student objectives on reading fluency and math were implemented (one in math and one in reading per student), as it was believed that improvements in these areas could contribute to increased overall achievement, decreased absenteeism, and reduction in emotional and behavioural difficulties. These individualized student objectives were met in the context of twelve sessions in a self-contained classroom setting.

#### Curriculum as usual groups

Contrary to regular curriculum classroom, special classes have more hours with their French teachers. They all receive math and English classes but have fewer other subjects then regular classes. They also have a lower student/teacher ratio than the latter.

### 3. Outcome measures

#### Primary outcome

The primary outcome was impairment from emotional and behavioural symptoms reported by youth. Impairment from emotional and behavioural symptoms was assessed by the Impact Supplement of the Strengths and Difficulties Questionnaires (SDQ) [32] completed by the adolescents. The SDQ is a 25-item Likert scale assessing emotional and behavioural symptoms. This questionnaire includes an impairment measure that enquires about symptoms in terms of chronicity, distress, social impairment, and burden for others. The SDQ has been translated into more than 20 languages and has been widely used in culturally diverse settings [33]–[35]. A choice of the French or English version was offered to the adolescents, and versions in other languages native tongue were available on request. The reliability and validity of the SDQ make it a useful measure of the emotional and behavioural difficulties and of the adjustment in youth [36].

#### Secondary outcomes

1) *Emotional and behavioural symptoms* were assessed by SDQ global scores (teacher and youth reports) and impairment assessed by teachers (impact supplement). 2) *School performance* was assessed on the basis of the first and the last report cards of the school year (T0 and T1). The first report is issued in November, before the beginning of the program, and the last one is issued in June. Because of school calendar, school performance measures were thus respectively anterior and posterior to T0 and T1 questionnaire administration. We considered students' grades in mathematics and French, the two compulsory subjects in special classes. It is to be noted that all the teachers were blind to the fact that school performance was part of the study assessment.

#### Control variables

The following socio-demographic variables were documented: gender, age, socio-economic status (family income and parents' employment status), country of birth of youth and of parents, ethnicity, years in Canada, language proficiency (English and French), migratory status (immigrants, refugee or citizen). In addition, contact with or use of mental health services outside of the school was noted.

### 4. Analysis

To assess the effect of the theatre and tutorship interventions on the impairment scores and the global scores, as reported by both teachers and students, we fit the data using linear mixed effect models which incorporate the correlation between students in the same class, due to the nature of the randomization of the interventions by classroom. We modelled each continuous outcome at follow-up using baseline measurement and treatment group as covariates. We included the treatment group as a covariate in order to measure the size of the effect of a particular treatment on the outcome scores. We then fit models including an interaction between baseline and treatment group, as well as models with covariates gender and interaction between gender and treatment group, and finally models with covariates baseline, treatment group, gender, baseline-treatment interaction, and gender-treatment interaction. In addition, we considered the effect of immigration status on the global and impact scores by running the models described above on subgroups of students based on first- or second-generation immigrant status.

Finally, to assess the effect of the theatre and tutorship interventions on math and French grades, we again used linear mixed effect models for the models on math and French grades to control for the heterogeneity within classes. We modelled the follow-up grades using baseline grade measurement and treatment group as covariates. We then included an interaction between baseline and treatment, as well as models with covariates gender and interaction between gender and treatment group, and finally the double-interaction model as before.

All analyses were done using the R statistical package, version 2.15.2. All models with SDQ Global scores or SDQ Impairment Impact scores as the outcome were modelled using the lme4 package. All linear mixed effect models run with SDQ Impairment Impact scores as the outcome were modelled under a Poisson family assumption because the impact scores only take on positive integer values.

## Results

The baseline characteristics of the subjects are shown in Table 1. There were 477 students in the study, with 140 assigned to the control group, 157 assigned to the theatre intervention group, and 180 randomized to the tutorship intervention. There were slightly more males than females in each intervention group. More than half of students in all groups had at least one working parent. Approximately 40% of students in each group were not born in Canada. The mean baseline self-reported SDQ global score reported was close to 11 for each group, while the mean baseline teacher-reported SDQ global score varied from 8.7 to 10.1 depending on the intervention group. The mean baseline self-reported SDQ impact score was <1 but quite variable, while the mean baseline teacher-reported SDQ impact score was >1 and had larger standard errors than the self-reported impact scores. There was no significant difference between the self-reported and teacher-reported scores at baseline.

**Table 1:** Baseline characteristics for each treatment group: (Mean +/− s.d. (% of missing observations) for continuous data or n (% of students who responded) for dichotomous data).

		Treatment Group		
Baseline Characteristics		Control (n = 140)	Theatre (n = 157)	Tutorship (n = 180)
**Sex**				
	**Female**	64 (46%)	64 (41%)	74 (41%)
	**Male**	71 (51%)	89 (57%)	102 (57%)
	**Missing**	5 (4%)	4 (2%)	4 (2%)
**Age**		13.99+/−1.22 (4%)	14.03+/−1.34 (3%)	13.57+/−1.11 (3%)
**Language Spoken at Home**				
	**French**	44 (31%)	52 (22%)	40 (22%)
	**English**	38 (27%)	30 (19%)	41 (23%)
	**Other**	52 (37%)	71 (45%)	93 (52%)
	**Missing**	6 (5%)	4 (3%)	6 (3%)
**Parents' Working Status**				
	**Father works**	86 (64%)	110 (72%)	127 (72%)
	**Missing**	36 (26%)	27 (17%)	32 (18%)
	**Mother works**	81 (60%	90 (59%)	102 (58%)
	**Missing**	17 (12%)	13 (8%)	19 (11%)
**Youth's Birth Place**				
	**Born in Canada**	75 (54%)	88 (56%)	99 (55%)
	**Not born in Canada**	59 (42%)	64 (41%)	76 (42%)
	**Missing**	6 (4%)	5 (3%)	5 (3%)
**Father's Origin**				
	**Canada**	11 (8%)	9 (6%)	13 (7%)
	**Asia**	31 (22%)	35 (22%)	57 (32%)
	**Mexico/Latin America**	14 (10%)	19 (12%)	23 (13%)
	**Europe/Russia**	11 (8%)	7 (4%)	11 (6%)
	**Middle East**	17 (12%)	28 (18%)	21 (12%)
	**Africa**	12 (9%)	9 (6%)	11 (6%)
	**Caribbean**	12 (9%)	9 (6%)	11 (6%)
	**Missing**	32 (23%)	41 (26%)	33 (18%)
**Mother's Origin**				
	**Canada**	14 (10%)	12 (8%)	13 (7%)
	**Asia**	31 (22%)	33 (21%)	59 (33%)
	**Mexico/Latin America**	17 (12%)	21 (13%)	23 (13%)
	**Europe/Russia**	10 (7%)	7 (4%)	4 (2%)
	**Middle East**	17 (12%)	28 (18%)	21 (12%)
	**Africa**	12 (9%)	12 (8%)	13 (7%)
	**Caribbean**	12 (9%)	12 (8%)	13 (7%)
	**Missing**	27 (19%)	32 (20%)	34 (19%)

A summary of the outcome measures according to treatment intervention is shown in Table 2. The self-reported SDQ global scores at follow-up are approximately 11 for each group, while the teacher-reported SDQ global scores at follow-up show greater differences between groups. The self-reported SDQ impact scores at follow-up are much less than 1 for all intervention groups, while the teacher-reported SDQ impact scores at follow-up are again larger than 1 for all groups with high variability.

**Table 2:** Summary of outcome measures for treatment group Mean +/− s.d. (% of students with missing scores/grades).

Outcomes		Treatment Group		
Primary Outcome		Control (n = 140)	Theatre (n = 157)	Tutorship (n = 180)
	Self SDQ Impact	0.48+/−1.18 (14%)	0.40+/−1.10 (7%)	0.53+/−1.27 (7%)
**Secondary Outcomes**				
	**Self SDQ Global**	10.98+/−5.09 (12%)	10.87+/−5.32 (7%)	11.47+/−4.42 (8%)
	**Teacher SDQ Global**	9.95+/−6.41 (6%)	7.88+/−5.77 (6%)	10.82+/−6.18 (6%)
	**Teacher SDQ Impact**	1.47+/−1.77 (6%)	1.14 +/−1.69 (8%)	1.29+/−1.52 (7%)
	**French Grade**	57.8+/−11.0 (20%)	62.2+/−11.9 (21%)	61.82+/−9.47 (32%)
	**Math Grade**	54.0+/−19.8 (20%)	59.1+/−16.2 (23%)	57.8+/−15.1 (32%)

Table 3 shows the results of the models on the self-reported score outcomes with covariates baseline scores, treatment group and baseline-group interaction. The estimates of the effect of the theatre intervention in all four models did not indicate a significant decrease of both the self-reported global scores and impairment scores. There is also a larger, significant decrease for those subjects with higher self-reported global and impact scores.

**Table 3:** Model Estimates (SE) for Self-reported Global and Impact Scores.

Covariates	Simple (Primary Outcome)	Interaction (Primary Outcome)	Simple (Secondary Outcome)	Interaction (Secondary Outcome)
**Intercept**	−1.38 (0.26)	−1.31 (0.27)	3.67 (0.57)	3.78 (0.95)
**Baseline Global**	−	−	0.66 (0.04)	0.65 (0.08)
**Baseline Impact**	0.40 (0.04)	0.38 (0.05)	−	−
**Theatre**	−0.12 (0.35)	−0.19 (0.38)	−0.43 (0.50)	−1.38 (1.24)
**Tutorship**	0.29 (0.33)	0.19 (0.36)	−0.20 (0.49)	0.50 (1.27)
**Base. Global: Theatre**	−	−	−	0.08 (0.10)
**Base. Global: Tutorship**	−	−	−	0.06 (0.10)
**Base. Impact: Theatre**	−	0.04 (0.10)	−	−
**Base. Impact: Tutorship**	−	0.06 (0.09)	−	−

Table 4 presents the results of the models corresponding to those in Table 1 for the teacher-reported global and impact scores. We see a similar pattern in the estimates, with one notable exception. While most of the estimates are not significantly different from zero, we note that the estimates of the effect of two interventions on the teacher-reported global scores in the interaction model are positive, indicating that the interventions seem to increase the global scores. However, the standard error is large which implies that this effect is quite variable.

**Table 4:** Model Estimates (SE) for Teacher-reported Global and Impact Scores.

Covariates	Simple (Primary Outcome)	Interaction (Primary Outcome)	Simple (Secondary Outcome)	Interaction (Secondary Outcome)
**Intercept**	−0.48 (0.17)	−0.30 (0.18)	3.32 (0.85)	2.12 (1.01)
**Baseline Global**	−	−	0.65 (0.04)	0.78 (0.07)
**Baseline Impact**	0.35 (0.03)	0.29 (0.05)	−	−
**Theatre**	−0.27 (0.22)	−0.71 (0.26)	−0.78 (1.06)	1.26 (1.34)
**Tutorship**	−0.07 (0.21)	−0.15 (0.24)	0.95 (1.05)	2.04 (1.39)
**Base. Global: Theatre**	−	−	−	−0.23 (0.09)
**Base. Global: Tutorship**	−	−	−	−0.12 (0.09)
**Base. Impact: Theatre**	−	0.18 (0.07)	−	−
**Base. Impact: Tutorship**	−	0.04 (0.06)	−	−

We considered whether gender had an effect on the outcome scores in both the self-reported and teacher-reported cases. Although the estimates are not presented here, we did not see a significant effect of gender on the global or impact scores. However, there was a slightly more decreasing effect of the theatre intervention for female subjects on the teacher-reported outcome scores but still not statistically significant.

The model estimates for the double-interaction models which include covariates baseline, treatment group, gender, baseline-treatment interaction and gender-treatment interaction are not presented here. Nearly all of the estimates are not statistically significant as in the gender models. There is evidence that overall, subjects with higher baseline scores may see a larger decrease in follow-up scores. However, the treatment group is not significant when compared to the models without the intervention covariate using ANOVA.

We fit the same five models including baseline, treatment group, gender and interactions as covariates to both the math and French grades. In all the models, for both math and French grades, there is no significant difference in either the theatre or the tutorship intervention groups compared to the control group. As there are no significant results in these models, they are not presented here.

Finally, in order to distinguish possible variations in the interaction effect according to migration stage, the simple and interaction models on both the global and impact scores (primary and secondary outcomes) were fit on subsets of students, based on first- and second-generation immigration status. Tables 5 and 6 present the results of the models on the self-reported impact and global scores for the first and second generation students respectively. When considering the interaction model for the self-reported impact scores, there is a small but significant negative effect of the theatre intervention among first-generation immigrants (a decrease in self-reported impairment), while there is a significant positive effect among the second-generation immigrant students (an increase in self-reported impairment). The theatre intervention has a non-significant negative effect on the global scores in both subgroups of students.

**Table 5:** Model Estimates (SE) for Self-reported Global and Impact Scores for First-generation Immigrant Students.

Covariates	Simple (Primary Outcome)	Interaction (Primary Outcome)	Simple (Secondary Outcome)	Interaction (Secondary Outcome)
**Intercept**	−1.19 (0.34)	−1.11 (0.34)	5.12 (0.85)	4.54 (1.25)
**Baseline Global**	−	−	0.52 (0.06)	0.57 (0.10)
**Baseline Impact**	0.34 (0.05)	0.30 (0.06)	−	−
**Theatre**	−0.54 (0.43)	−0.97 (0.49)	−0.85 (0.77)	−1.08 (1.77)
**Tutorship**	0.33 (0.41)	0.24 (0.44)	0.23 (0.75)	1.96 (1.73)
**Base. Global: Theatre**	−	−	−	0.03 (0.15)
**Base. Global: Tutorship**	−	−	−	−0.15 (0.14)
**Base. Impact: Theatre**	−	0.44 (0.20)	−	−
**Base. Impact: Tutorship**	−	0.06 (0.11)	−	−

**Table 6:** Model Estimates (SE) for Self-reported Global and Impact Scores for Second-generation Immigrant Students.

Covariates	Simple (Primary Outcome)	Interaction (Primary Outcome)	Simple (Secondary Outcome)	Interaction (Secondary Outcome)
**Intercept**	−2.39 (0.46)	−2.41 (0.53)	2.76 (0.88)	3.02 (1.69)
**Baseline Global**	−	−	0.77 (0.06)	0.74 (0.14)
**Baseline Impact**	0.40 (0.07)	0.45 (0.47)	−	−
**Theatre**	1.14 (0.52)	1.38 (0.59)	−0.26 (0.74)	−1.17 (2.09)
**Tutorship**	0.82 (0.55)	0.58 (0.63)	−0.56 (0.72)	−0.29 (2.10)
**Base. Global: Theatre**	−	−	−	0.08 (0.17)
**Base. Global: Tutorship**	−	−	−	−0.02 (0.17)
**Base. Impact: Theatre**	−	−0.19 (0.48)	−	−
**Base. Impact: Tutorship**	−	0.14 (0.48)	−	−

## Discussion

The main finding was that the theatre intervention was not associated with a greater reduction in self-reported impairment and symptoms in youth placed in special class because of learning, emotional and behavioural difficulties than a tutoring intervention or a non-active control group. Although the estimates of the different models show a decrease in both self-reported and impairment scores in the theatre intervention group, this was not significant for the overall group. However, the difference in the effect of the drama workshops on impairment for first and second generation adolescents suggests that the intervention may support the adaptation of newcomers, while, in an educational setting which may reinforce feelings of exclusion, it seems to exacerbate the perception of dysfunction in second generation youth. The difference between the population studied previously and the sample of this trial partially supports this hypothesis. The theatre workshops had previously been studied with classes of very recently arrived immigrants and refugees, which included youth with some learning, behaviour and emotional difficulties [22]. A pilot study also showed promising effects for classes of newcomers who presented a significant academic delay [23]. In both of these studies the youth were experiencing a rapid transition in culture and environment and the theatre workshops appeared to help them regain a sense of mastery and work through their past traumas and losses. In the present sample, half of the youth were second generation immigrants and they clearly had a different response to the drama workshops. It could be that in this particular group the difficulties are overall of a more chronic nature and that the changes brought forward by a creative expression intervention, although maybe allowing an awareness of their difficulties and frustration, do not, in the short term, constitute a tool for personal transformation. This would however need to be verified.

The absence of effect of the tutorship intervention is surprising but in line with Wilson and Lipsey's [2] meta-analysis which documents only marginal effects for school-based interventions for aggressive and disruptive behaviours in special schools and classrooms. The authors suggested that this relative lack of impact may reflect the fact that the usual programing of these special settings already targets adequately the youth difficulties. It is however also possible to think that these special settings are demanding and sometimes exhausting for the teachers who may become less invested and less engaged with their students. Knowing that teacher engagement is a key factor for the academic engagement and achievement of newcomer youth and minorities [37], [38], this may point to the existence of eventual systemic problems which a time limited intervention cannot address.

The baseline teacher reported Impact Scores are above the SDQ impact borderline cut-off of 1, indicating that overall these youth present serious levels of impairment. It is noteworthy that after intervention, the teacher-reported global scores increased significantly while the impairment scores remained unchanged. Because the teachers were not blind to the experimental status, this could reflect the fact that they had become more aware of the level of distress of their students after the intervention, rather than an actual increase in symptoms, which would probably have been associated with a deterioration of functioning and with some teachers' complaints about the intervention, which was not the case.

In the present study, the fieldwork was very difficult and both the theatre and the tutorship intervention teams needed regular support and encouragement because they were experiencing and witnessing disrespect and sometimes bullying from the students. This led the teams to address basic physical and emotional safety issues during the intervention. In such a disrupted environment, there may have been only limited opportunity for each student to address issues relevant to him or her and they may not have felt safe to do so. Overall, it is possible to envision that when institutional structures are not able to provide a secure enough space, alternative interventions may have a more limited impact because other structural changes are required before a modification of the curriculum could be effective.

In conclusion, in this trial a twelve-week theatre intervention was associated with a small improvement of impairment in first generation immigrant youths with EBD placed in special education classes and with an increase in impairment reported by second generation youth. These results question the potential usefulness of these interventions in those particular settings. They suggest that the difference in first and second generation response to classroom intervention needs to be examined more thoroughly. They may also suggest that these educational settings present particular challenges and may require interventions which specifically address some of the organizational dimensions.

## Supporting Information

Checklist S1 CONSORT Extension for Cluster Trials Checklist.
(DOCX)

Protocol S1 Study Protocol.
(DOCX)
