# Supplementary material for: A Cluster Randomized-Controlled Trial of a Classroom-Based Drama Workshop Program to Improve Mental Health Outcomes among Immigrant and Refugee Youth in Special Classes
Source: PLoS One. 2014 Aug 15;9(8):e104704. doi: 10.1371/journal.pone.0104704 (PMC4134233; doi:10.1371/journal.pone.0104704)
Supplement: Protocol S1 — Study Protocol. (DOCX) [file pone.0104704.s002.docx]

**1. THE NEED FOR A TRIAL**

**1.1 What is the problem to be addressed?**

Many immigrant and refugee families, have experienced organized violence in their homelands and

many suffer from poverty, often living in unstable environments (1-10). In spite of their high exposure to adversity, immigrant and refugee families persistently underutilize mental health services(11, 12) and often feel threatened by targeted interventions offered through the schools because of the potential stigma for the child and family (11, 13, 14). A review of the evidence supporting school-based intervention suggests that programs focusing on promoting mental health and coping are more effective than attempts to prevent mental health problems (15, 16). This consideration may be even more important in the case of multiethnic minority communities, whose conception of mental health may differ from that of the host society and who may experience intervention as another form of exclusion. Schools, which are the main point of contact between these children and the host society, are in a key position to implement prevention programs based on ecological principles that enhance young people’s ability to adapt to their new lives (17-21).

Although the role of schools in preventing emotional and behavioural problems and promoting immigrant and refugee mental health is increasingly recognized (19, 22-26), few programs have been designed and evaluated for schools located in multiethnic neighbourhoods. In Montreal, where more than 50% of children and youth are first and second generation migrants, the need to develop such preventive programs has been identified by a number of schools. Setting up a classroom program to promote mental health and coping in newly arrived immigrant and refugee children presents several challenges. First, the population is heterogeneous, both culturally and in terms of experiences in their homeland and during migration. Furthermore, the gap between school and family is wide, and a program devised by host country therapists or educators could easily become just one more disparate element in the children’s two separate worlds (27). Last, despite many small-scale innovative projects, little is known either in theory or in practice, about the types of activity that may work best for children from different cultural backgrounds (28-31). The last decade has seen the emergence of more systematic evaluative research in the field of mental health programs for children of war (32-36) and for immigrant and refugee children (37). It is to be noted that gender appears to be a key variable when assessing the effect of different types of intervention, whether psychotherapy, creative play or mixed modalities, with boys responding usually less to the proposed interventions (36, 38).

Building on the literature describing the usefulness of creative expression programs for immigrant and refugee children in clinical and community settings (27, 39-42), our team composed of Montreal schools, community organizations and health professionals developed a set of theatre-based prevention programs for preschools, elementary and high schools. The aims of the programs is to help newly arrived children and adolescents bridge the gap between home and school, past and present, as well as to work through experiences of loss and trauma (8, 43-47). Through repeated qualitative evaluations from the pilot stage of the program, four key elements associated with the effects of the programs were identified: constructing a safe space, acknowledging and valuing diversity, establishing continuity, and transforming adversity (8). A quantitative evaluation (experimental vs. control) of three programs (preschools, elementary and high schools) showed that all of them had a significant effect on child and youth mental health. At the elementary level they decreased children’s level of emotional and behavioural symptoms (48). In high school, they decreased impairment and improved school performance, in particular for boys (49). Finally, in preschool, the evaluation results provided evidence

that the creative expression workshops decreased significantly the global symptoms reported for the experimental group children (50, 51).

**Theatre intervention for adolescents with behavioural and learning difficulties**

Schools asked for the theatre program, originally developed for high schools, to be adapted to suit young

immigrants enrolled in a special class for students with learning and behavioural problems and an alarming rate of serious mental health problems. Studies of North American and European populations have long highlighted the overlap (from 25 to 75% depending on the study) that exists between learning problems and emotional and behavioural problems — a phenomenon often described in terms of comorbidity (52-55). With regard to treatment programs, a meta-analysis found that while academic support is the most effective form of assistance at the elementary level, at the secondary level tutoring groups for students with learning problems achieve the best outcomes (56). These groups are designed, however, for young people who share the same cultural background, the same values and the same view of the world. In 2003–2004 in Quebec, the proportion of immigrant students classified as “handicapped or with adaptation or learning problems” was comparable with the proportion for all students (57). Given the views that their families and communities associate with being assigned to a special class, and given the social position attached to minority cultural status in economically socio-disadvantaged neighbourhoods, these children often experience a double exclusion (58). In the field of ethnic studies, the ethnic and racial biases underlying referral to special classes have been studied extensively in the United States over the last 20 or 30 years, yet recent studies have also highlighted the adverse effects of segregation from peers having weak academic performance for minority group children, who are over- represented in disadvantaged schools (59-61). Parents in these cultural communities fear stigma and often perceive specialized services and classes as a threat (62, 63). The lack of understanding and the prejudices that exist between immigrant communities and host country institutions cause problems with respect to diagnosis (64) and the therapeutic measures that should be given preference. Treatment programs have been reported to sometimes accelerate the marginalization of these young people, further aggravating problems and increasing service deadlock (65). Under these circumstances, it is essential to weigh the pros and cons carefully before proposing the introduction of a wider array of specialized individual services which, though aimed at real problems, do not address the power imbalance that exists between minority communities and schools and may in fact reinforce, rather than transform, the perception schools have of these communities (66). On the other hand, a consolidation of identity roots through school support programs seems to be associated with an empowerment of these young immigrants and with a subsequent improvement in their learning (67).

For adolescents, the opportunity to express and become comfortable with multiple identities simultaneously is one of the keys to the transforming power of theatre (68). In a school context, theatrical expression fosters social development, the sharing of responsibilities and team-building (69,

70). Theatrical expression also facilitates non-verbal expression for young people who are struggling with limitations in this respect, and provides an opportunity for acting out conflicts and exploring various possible solutions in a safe environment. Playback theatre is a theatrical movement that aims to achieve personal and social transformation through sharing a theatrical experience within a ritual space (71-73). It is based on a number of committed theatre movements, that as described by Moreno (74) seek to reach out to silent, isolated individuals who see themselves as, or are perceived as, being different. Playback theatre program promotes a role of subject for the marginalized and the excluded that can give them back a certain amount of power over themselves and their environment (75-77).Used in many different countries, it has been found to be an effective instrument for working with young people

who have behavioural problems (73). While the use of drama therapy for young immigrants has primarily been documented clinically (78), its effects have only rarely been evaluated quantitatively. A randomized trial on drama therapy in a non-multiethnic school environment suggests that it is more effective than other group treatment modalities and shows that its effects are still present one year after the end of the program (79). In a multiethnic environment, the non-verbal, metaphorical characteristics of this type of intervention facilitates its acceptance and cultural appropriateness (78).

**Impairment as a measure of outcome**

Functional impairment is a characteristic of the individual that indicates in a global way how the

individual functions across life’s roles (80). The assessment of impairment, as opposed to measurement of disorder or symptom severity, is important for four main reasons: 1) A consortium of health care administrators, clinicians and parents have emphasized the centrality of ‘improvement in functioning’ in the assessment of treatment outcome. Outcomes such as an adolescents’ ability to live at home with their families, to attend school, and to function competently in social situations benefit adolescents and their families as well as contribute to the public good. The consumer perspective places a high value on functional outcomes because functional impairment creates more hardship for families and society (81).

2) Functional impairments may resolve more slowly than recovery from a disorder itself, so information on the youth’s adaptive functioning is needed to assess and facilitate full psychosocial recovery (82, 83).

3) Outcomes research has demonstrated that psychosocial improvements do not necessarily correlate with symptoms resolution of major psychiatric disorders (83-85). 4) The increasing demand for evidence-based treatments emphasizes outcome in multiple domains of functioning. Thus, the definition of treatment success should consider adolescents’ return to prior functioning or optimizing their functioning, not just amelioration of their symptoms or remission of their disorder. Scales measuring functional impairment can elucidate the impact of illness on youths, identify targets for treatment, determine service needs, and monitor treatment effectiveness. These scales are widely used in community mental health and health service delivery, where they assist in providing evidence-based treatment. Evaluating outcomes of intervention for adolescents with emotional and behavioural difficulties is by no mean an easy methodological choice. Although many studies have focused on the reduction of symptoms, more and more it appears that the level of impairment perceived by the youth himself better reflect changes in functional capacities (86). It is to be noted that the evidence about efficacy of school based programs for refugee children is mostly based on symptom scores (14, 34, 36,

87). Impairment is rarely measured in spite of the well known discrepancy between social adjustment (functioning) and symptoms in this population (23). Although there is overall a paucity of data linking changes in impairment scores to clinical significance for the youth, the literature indicates that it may be more clinically meaningful than the shift in symptoms scores (88). In our pilot study, the fact that the significant change in impairment was associated with a significant improvement of academic performance supported this last hypothesis (49). These results were replicated in a recent pilot in classes for underschooled youth (89). For these reasons although we will monitor, as a secondary outcome, the decrease in symptom scores, we have chosen to consider the impairment as reported by adolescents as the primary outcome measure.

**1.2 Principal research questions to be addressed:**

**Aim:** To evaluate the efficacy of a school based theatre intervention program for immigrant and refugee

youth in special classes in improving mental health and academic outcomes.

**Primary hypothesis:** Students in the theatre intervention group will report a greater reduction in

impairment from symptoms (emotional and behavioural) compared to students in the control groups from pre-intervention (T0) to the end of the 12-week intervention program (T1).

**Secondary hypothesis #1:** Students in theatre intervention group will report a greater reduction in impairment compared to student in the control groups from T0 to T2.

**Secondary hypothesis #2:** Students in theatre intervention group will report a greater reduction in impairment compared to students in the tutoring group from T0 to T1 and T2.

**Secondary hypothesis #3:** Compared to students in the tutoring and control groups, students who received the theatre program intervention will report a greater decrease in global emotional and behavioural symptoms as reported by themselves and by their teachers from T0 to T1 and T2.

**Secondary hypothesis #4:** Compared to students in the tutoring and control groups, students who received the theatre program intervention will report a greater reduction in impairment (teacher reported) from T0 to T1 and T2.

**Secondary hypothesis #5**: Compared to students in the tutoring and control groups, students who received the theatre program intervention will report a greater increase in the quality of peer relationships from T0 to T1 and T2.

**Secondary hypothesis #6**: Compared to students in the control groups, students who received the theatre program or the tutoring intervention will report a greater improvement in school performance.

**1.3/1.4 Why is a trial needed now?**

Theatre therapy for teenagers is part of set of prevention programs developed in partnership with schools

(Sand Play Program at the preschool level, Arts and Stories at the elementary level, and Theatre at the secondary level). Qualitative evaluation of theatre expression workshops in classes for newly arrived migrants (French as second language) suggests that workshops are a safe expression space in which adolescents benefit from the support of their peers, the team and the ritual nature of the activity. The workshops promote the development and assimilation of the different transitional experiences associated with adolescence, migration and having a hybrid identity, and enable young people to transform, their experiences of adversity into a learning opportunity (Rousseau et al., 2005). The results of the quantitative evaluation, which compares students in welcome classes randomly distributed between experimental and control groups (n=123), revealed a significant reduction (p < 0.05) in the perception of distress and impairment associated with mental health symptoms experienced by adolescents who took part in the activity compared with adolescents in the control group (*ß* = -0.194). With respect to academic performance, results indicated a significant improvement (p < 0.05) in performance in math among the adolescents who took part in the activity compared with those in the control group (*ß* = -

0.167) (49). The teachers in the experimental groups also reported an increase in the general participation level of their students in class compared with the teachers in the control group.

In the wake of this initial research project, pilot projects in special classes incorporated the following modifications into the workshops: (1) simplification of the verbal presentation of the activities; (2) increased involvement of the students in musical improvisation as a means of channelling certain behaviours; (3) refocusing of the themes on subjects of interest to less educated students. The results of the pilot projects (2005 to 2009) confirmed the benefits of these changes. More than in regular classes, the workshops transformed the teachers’ perception of the adolescents by highlighting not only the students’ experiences of adversity (trauma, grievance, discrimination), of which the teachers were often unaware, but also their resources and strengths, which are often not recognized in the usual school context (90). On the basis of these results, a number of Montréal’s high schools submitted a request asking for the program to be adapted to suit young immigrant students who had been assigned to a

special class because of learning and behavioural problems. This present project builds on the previous work in three ways: (1) This will be the first randomized trial of the use of theatre as a means of helping young people who are struggling with learning and behavioural problems; (2) Past trials did not include a follow-up measure owing to budget and time constraints related to the funding received because of the difficulty in following up with students in classes for newly arrived immigrants, a very mobile population. It is important to determine to what degree the therapy has an effect over time; (3) Past protocols did not include a measure of a comparison program to control for the increased attention received by the youth during the intervention. This is important because recent school based RTC’s of CBT interventions have shown that non specific factors, and in particular positive reinforcement, may play a key role in the students’ improvement (91).

**1.5 How will the results of this trial be used?**

With respect to school practices in multiethnic and disadvantaged neighbourhoods: (1) the program will

help expand and diversify the teaching methods for reducing problem behaviours and improving social adjustment and academic performance of these adolescents; (2) the project will propose group intervention modalities for young people with learning and behavioural problems, organized around three key concepts that underlie the theatre expression workshops: creativity as a means of transforming experience, empowerment through group discussion forums, symbolic and real consolidation of social bonds by building solidarity through shared experiences. These group intervention modalities could complement and, in some cases, replace certain individual treatments that, owing to their potentially stigmatizing nature, are often perceived with distrust, or even avoided, by immigrant families; (3) the project will also confirm the benefits of an integrated approach that involves providing mental health services in partnership with schools when it comes to developing culturally appropriate prevention programs. Transfer of knowledge will rely on dissemination strategies developed by the team to support teachers’ implementation of the creative workshops in regular and welcoming classes (92). School partners and education and health decision makers will be regularly informed through the advisory committee. Preliminary results will be discussed through videoconference at the local, provincial and national levels. A provincial workshop and a national seminar, bringing together representatives of schools and health services will be organised the last year.

**2. THE PROPOSED TRIAL**

**2.1 What is the proposed trial design?**

The experimental method we are proposing is an open trial in the course of which three experimental

conditions will be compared: (1) Participation in the theatre program; (2) Participation in a group tutoring activity; (3) Participation in the regular special class school curriculum without these activities.

**2.2 What are the planned trial interventions?**

2.2.1 Drama workshop program:

**Intervention program objectives**

The purpose of the theatre expression program adapted to special classes for students with learning and

behavioural problems is to facilitate the reappropriation and sharing of group stories by young immigrants and refugees in order to (1) give support to the construction of meaning in an individual’s personal history; (2) foster the grieving processes associated with immigration (separation, transition, loss of expectations) and experiences of academic failure; (3) help consolidate multiple identities; (4) enhance appreciation of differences and the development of resistance strategies without increasing marginalization and exclusion, in order to:

Minimize the distress and behaviours stemming from the losses of migration, the tensions of belonging to a minority, and learning and behavioural problems identified by the school.

Foster social adjustment and academic performance of adolescents.

Provide schools and teachers with tools for responding to emotional and social needs of these young people by adapting teaching methods to suit their specific experiences.

**Description of the drama workshop program:**

The theatre expression workshops will run for 12 weeks, with one 90-minute workshop per week (only

the contents of the user’s manual, teacher training version, is attached, because of space constraints) (Appendix 1). They will be incorporated into the regular class timetable and will be run by the two members of the intervention team who have training in the theatre and psychology, and the homeroom teacher, whose level of direct involvement will increase gradually as he or she becomes familiar with the workshops. The program is based on playback theatre in the following respects: (1) the purpose of the workshops is to create a safe, respectful environment, in which possible tensions can be explored in the knowledge that they will be contained by the group; (2) artistic performance is not one of the objectives; what is expressed remains within the group and is not presented as a show; (3) the theatrical structure is that of playback theatre: a play director coordinates and contains the story as it unfolds; he or she directs a group of actors (the workshop team and some students) and musicians (students) as they play out a story. Each workshop is structured in more or less the same way. The workshop leaders act as guides and encourage the participants to improvise verbally, musically and through gestures, and to explore alternative scenarios. The workshops are structured on the basis of four types of activities:

1. Warm-up: generally for the first two meetings (sessions 1 and 2), exercises focussing on listening, trust and non-verbal improvisation help participants get to know each other while encouraging play, humour and imagination. The warm-up exercises are done in a respectful, tolerant environment that allows for individual differences while building group solidarity.

2. Improvisation: Theatrical methods (fluid sculptures and ambivalent situations), inspired from youth personal stories related to a theme, are used to familiarize the participants with improvisation. Later, full stories contributed by the adolescents are explored, played out, validated and played out again in a search for alternative scenarios, for example, when they have a distressing outcome, demonstrate a lack of emotional regulation or illustrate a situation of exclusion, discrimination (Boal transformation). These alternative scenarios are developed collectively and contribute to personal and group empowerment. Over the course of the 12 workshops, a variety of topics are explored. In the program adapted to the needs of special classes, we focus on the theme of belonging and exclusion (four sessions: 3 to 6) and on the different aspects of learning (especially the learning that comes from personal experience, even adverse experience) (four sessions: 7 to 10). We also touch on the topics of family and friends, which connect with questions of solidarity and social network, and the topic of transitions, which evokes migration, adolescence or other turning points in the youths’ experience.

3. The last activity (11-12) is called a “story house” and involves group work around stories which have been written by the youth and posted on the walls. It represents the diversity and richness of the youth’ experiences and their personal and collective capacity to transform adversity.

**2.2.2. Group tutoring program:**

The theatre workshops will be compared with a program in the context of a self-contained classroom.

Students with emotional and behavioural difficulties (EBD) have been found to have comorbid academic and behavioural challenges (93, 94). Irrespective of the classroom placement (special or general education classes), the interventions for students with EBD almost exclusively focus on

behavioural management with academic learning being addressed secondarily, if at all (95). These students are often achieving significantly below level as compared to students without disabilities and exhibit underachievement in all academic areas (96). Research also consistently indicates that they do not improve over time and that in some areas (e.g., math) their difficulties increase (see review, (97)). Some subtypes such as externalizing behaviour and attention were more closely associated with academic underachievement. In addition, high school students with EBD in a self-contained setting indicated that overall school adjustment was predictive of academic performance (e.g., (98)). These interventions focused on targeting in-class academic skills associated with attention to the assigned task, good working habits, developing listening skills and academic reading in the classroom setting (93, 98-

100). Therefore, the comparison intervention of the proposed study is an academic in-class intervention that focuses on differentiated academic instruction and improved overall academic adjustment. Each self-contained class will include a core teacher who teaches curricula based on the Quebec Education Program with age appropriate competencies in reading, math, social studies and science content areas. In each classroom assigned to the tutorship intervention, two academic resource assistants will provide weekly in-class support to students for the same length of time than the drama workshop. Individualized student objectives on reading fluency and math will be implemented (one in math and one in reading per student), as it is believed that improvements in these areas will contribute to increased overall achievement, decreased absenteeism, and reduction in emotional and behavioural difficulties. These individualized student objectives will be met in the context of twelve sessions in a self-contained classroom setting.

**2.3 What are the proposed practical arrangements for allocating participants to trial groups?**

The target population for the intervention is made up of secondary school students attending a high

multiethnic density school who have been put in special classes, because of learning or behavioural problems. These classes are comprised of youth who typically have at least a 2 years academic delay. One third of the classes are composed of youth who have not finished their elementary school curriculum. A second third is composed of 7th graders and the third of 8th graders. Some years there may be a few classes of 9th grade. The students are 12 to 17 years of age. The five schools (Saint-Luc, Lucien Pagé, Antoine-de-Saint-Exupéry, La Dauversière and Saint-Henri) taking part in the program and its evaluation were selected on the basis of a long-standing partnership in these projects and because they serve disadvantaged, highly multiethnic communities. These high schools have a large number of special classes: 18 at Lucien Pagé, 16 at St-Luc, 9 at Antoine-de-Saint-Exupéry, 11 at La Dauversière and 9 at Saint-Henri (total: 63) (see table 2). Ten special classes will take part in the theatre program, ten in the group tutoring activity and ten others will constitute a control group. Students who decline to

participate in the research will nevertheless attend the workshops that are part of the class’s regular

curriculum, but they will not participate in the data collection. The ideal evaluative research design would have required that all students who agree to take part in the research project be distributed randomly among the experimental and control groups. Constraints imposed by the functioning of the secondary schools do not allow this, and our evaluative strategy is therefore based on school stratified random assignment to experimental or control status. In an initial stage, a list of the special class homeroom teachers will be drawn up and will form 5 sampling pools, one for each high school. In a second stage, 2 experimental classes, 2 tutoring classes and 2 general curriculum classes will be chosen randomly from each of the 5 sampling pools/schools for a total of 30 classes.

**2.4 What are the proposed methods for protecting against other sources of bias?**

The questionnaires will be filled in by the students themselves in class and by teachers who are not the

homeroom teachers. The teachers who fill in the questionnaires for each student will thus not be present in class with the students for the activity. Even if it cannot be guaranteed that these teachers will be blind to the experimental or control status of the students, given that the students can comment on their activities, they will not be involved in the activities. Lastly, educational achievement is an unbiased source of evaluation, as school marks are given by several different teachers and therefore do not depend solely on the teacher who is in attendance during the activity. At T2, all the teachers involved will be completely blind to the experimental status of the youth. Contamination is another possible bias. The theatre intervention is however quite complex and in our experience has never been implemented spontaneously by teachers who were willing to try it, after discussing with their colleagues. In the case of the tutoring intervention, the added manpower (two resource teachers) is something every teacher in special class would wish to have, but cannot implement independently.

**2.5 What are the planned inclusion/exclusion criteria?**

All students who are assigned to special classes based on behavioural or learning problems in grades 7-

10 in the participating schools will participate in the program and be included in the study if they and their parents consent to in the research. The natural classroom setting may include non-immigrant youth but they never represent more than 5% of the students in a particular class and they can also benefit from the workshops. The exclusion of more severe cases, although it facilitates the implementation of the intervention (34, 36), does not represent the everyday challenges that teachers encounter in special classes. A real classroom setting provides a better measure of effectiveness (88).

**2.6 What is the proposed duration of treatment period?**

The theatre expression workshops and the tutoring programs will both run for 12 weeks, with one 90-

minute session being held each week.

**2.7 What is the proposed frequency and duration of follow-up?**

Data on the evaluation of the effects of the theatre expression workshops on the students will be

gathered pre- and post-intervention, as well as at follow-up nine months after the end of the intervention. Whenever feasible, the evaluation of the maintenance of the effect of psychosocial interventions for some time (classically 6 months) after termination is a good indicator of their efficacy (34, 36). In the present case, a six month follow up would have resulted in a T2 measure in September, at the beginning of the next school year. This would not have enabled us to assess the medium term effect of the intervention on school performance because the first report card is in November. Furthermore, the teachers’ report Strength and Difficulties Questionnaire (SDQ) is more valid when teachers have been working with the youth for a few months. For these reasons we decided that T2 would be taking place at the beginning of December, 9 months after the end of the intervention. The evaluation questionnaires for each measurement time will be filled in during a 90-minute class period and will be designed so that students with learning difficulties can complete them in approximately 45 minutes. Theoretically all the students taking part will have another school year to finish (high school graduation is at the end of grade 11 in Quebec). However, the youth older than 16 years old who present behavioural and academic difficulties are more at risk to drop out of school than their peers. Nonetheless, in our past studies, the losses attributable to follow-up during a school year have been minimal (< 5%). We therefore do not expect a significant attrition at T2. Special measures will be taken to keep contact with the students who may leave the school at the end of academic school year. Families, who have recently immigrated, are quite mobile. To address potential attrition linked to moving, youth will be asked at T1 if they plan to remain in the same school and/or to move and if so, what is the name of their future school and/or there

new address. Permission to contact them in the future will be requested and contact information for the student will be obtained.

**2.8/2.9 Proposed primary and secondary outcomes measures and their measurement follow up**

The effect of the program on student outcomes, as evaluated by the students and the teachers, will be

measured at the three times by means of a self-report questionnaire. Impairment associated with emotional and behavioural symptoms, intensity of emotional and behavioural symptoms and the social adjustment of the youth will be measured by two informants (youth and teacher) following three main principles: (1) complementarity between the teacher’s perspective and the student’s; (2) maximizing the transcultural validity of the instruments; (3) opting for instruments with robust psychometric properties, given the heterogeneity of the sample.

**Primary outcome: Impairment of emotional and behavioural symptoms reported by youth.** Impairment of emotional and behavioural symptoms will be assessed by the Impact Supplement of the SDQ (101) completed by the adolescents. The SDQ is a 25-item Likert scale assessing emotional and behavioural symptoms. This questionnaire includes an impairment measure that enquires about symptoms in terms of chronicity, distress, social impairment, and burden for others. The SDQ has been translated into more than 20 languages and has been widely used in culturally diverse settings (4, 102,

103). A choice of the French or English version will be offered to the adolescents, and a version in their native tongue will be available on request. The psychometric properties of the SDQ are good (104). In our study, the internal consistency of the self-report Impairment was excellent (alpha = .96).

**Secondary outcome:**

1) Emotional and behavioural symptoms will be assessed by the SDQ global scores (teachers and youths

reports). In our study, internal consistency of the self-report version was satisfactory, although not high, for the total SDQ.

2) Impairment (teacher report) will be measured by the SDQ. The internal consistency of the teacher’s version was high (alpha = 0.91 at T1 and 0.90 at T2).

3) Relations with peers will be assessed using the Adolescent Friendship Inventory (AFI) designed by Rubenstein et al. (1989). This 30-item instrument requires the adolescent to indicate to what degree, on a scale of 1 to 5, the feeling expressed by the statement corresponds to his or her personal feelings in relation to his or her network of peers. It is reliable with adolescent refugees (alpha = .88) (105).

4) School performance will be assessed on the basis of the first and the last report cards of the school year (T0 and T1) and with the first report card of the subsequent year (T2). The first report is issued in November, before the beginning of the program, and the last one is issued in June. We will consider students’ grades in mathematics and French, the two compulsory subjects in integration classes. It is to be noted that all the teachers will be blind to the fact that school performance is part of the study assessment and that the mathematics teachers who will not participate in the intervention will also be blind to the experimental versus control status of their students (although schools are small milieu and he may be aware of the fact that some classes are receiving intervention, it is unlikely that he or she will distinguish the type of intervention which is going on).

**Control variables:**

The following socio-demographic variables will be documented: socio-economic status (family income

and parents’ employment status), country of birth of youth, country of birth of parents, ethnicity, years in Canada, language proficiency (English and French), migratory status (immigrants, refugee or citizen). In addition, contact with or use of mental health services outside of the school will be noted. A measure assessing the learning environment will allow us to control for the variance related to the

teacher and the school environment (inter-school variations). We will use the WIHIC (What Is Happening in this Class) questionnaire, developed by Aldridge and Fraser (106), to evaluate student cohesiveness, teacher support, involvement, task orientation, cooperation and equity in the class group. In a comparison with other learning environment measurement scales, it proved to be the instrument that provided the most explanation of inter-environment variance (107).

**2.10 Cost Analysis**

The research will document the physical resources required to carry out the two workshops: theatre

program and tutorship intervention. This will allow us to give an estimate of the costs to school boards interested in implementing the program. The total direct costs and expenses related to the music instruments and theatre material will be calculated as will the costs related to the hiring of professionals for the workshops. These will include the time spent by specialized staff (n=2) (ex: 2 staff members x

12 weeks x 1.5 hours 30$/h) for the intervention as well as any additional time required for training and supervision of teachers whose class was randomised into the two treatment arms. Any time teachers spent outside school hours for training regarding the workshops will also be measured (hours spent 27$ per hour (average teacher’s salary during the fiscal year).

In order *“to inform “value for money” judgments about an intervention or program”* (108), an economic evaluation assessing the ‘incremental change’ (109) will be carried out. The evaluation will compare the costs and effects of the theatre program to the costs of the tutorship intervention and to the

‘do nothing’ arm and this for the two time periods: T0-T1 and T0-T2. In order to choose the best program or intervention one has to weigh only the “incremental changes” in costs and consequences between the alternatives being compared and therefore it is not necessary to include all possible costs and consequences of each alternative (110). The costs that will be included in the analysis, as described above, are therefore complete. The Canadian guidelines for the economic evaluation of health technologies (111) state that in the denominator of the ratio one should use a valid outcome measure that is most important to the health of the person. In our context, the reduction in impairment from emotional and behavioural symptoms in students is of great relevance. The incremental cost- effectiveness ratio (ICER) will be calculated using all the expected costs and expected effects for the classes undergoing the theatre program and those with the tutorship and regular class such that:

ICER =

*tp*

*ts*

ICER =

*ts rc t t*

ICER =

∑ *E Ct*

*tp*

*E Ct*

*ts*

*ts rc t*

∑ *E Eff t*

*E Eff t*

*tp = theatre program rc = regular class ts = tutorship

where E(C*t*)*theatre program* = the expected cost with the theatre program in the 12 week time period *t* (T0 – T1), E(C*t*)*tutorship* = the expected costs with the tutorship in the 12 week time period *t* (T0 –T1), E(C*t*)*regular class*= the expected costs with attending regular class in the 12 week time period *t* (T0 –T1), E(Eff*t*) *theatre program* / *tutorship* / *regular class* = the expected effectiveness with the theatre program, tutorship and regular class, respectively, corresponding to the change in SDQ symptoms in the specified time

period *t* (T0 – T1 or T0 –T2), because we will be evaluating symptoms at pre (T0), post (T1) and 9 months later (T2). These cost-effectiveness ratios will produce the cost for each unit change in student level of impairment which quantifies for decision makers the trade-off between costs and effects associated with these programs (112). In this context, we will consider the cost for an average change of

3.95 points in SDQ which represents a moderate effect size (d = 0.50) improvement in impairment.

**2.11 What is the proposed sample size?**

Ten classes per condition will be included in the study, with an estimated 18 students per class. Power

calculations were performed assuming: 1) a compliance rate of 80%; 2) 5% loss to follow-up at the end of the twelve-week program (T1); 3) within-class variance and residual variance estimated from the pilot data.

Power calculations were performed via simulation, using the above parameters and assuming a Normal distribution of change in SDQ scores over a range of possible effect sizes. Accounting for the within- class clustering of children using a linear mixed effects model with a random effect for class and a fixed effect for the intervention, the proposed sample size has 77.5% power to detect a moderate effect size (d

= 0.50), and 84.5% power to detect a 5 point difference in SDQ score between the theatre intervention and the control group. In the pilot study, there was a mean difference in change on the SDQ of 5.85 points between the intervention and control groups. However, the study participants were newly arrived immigrant and refugee youth who had been undergoing recent intensive stress. The type of symptoms tends to decrease relatively rapidly with time, even if the interactions significantly helped them to subside (113). In the present study, we will be working with youth presenting emotional and behavioural difficulties for a relatively long time, since this prompted their inclusion in special class. We thus expect that these chronic problems, although possibly more severe, may be more difficult to shift. Studies of psychosocial interventions are typically powered based on a moderate effect size (e.g., d = 0.50) to represent clinically significant change (e.g., (114)), Depression: management of depression in primary and secondary care. Clinical practice guideline No. 23. London: National Institute for Clinical Excellence). Thus, the study was powered to achieve at least a moderate (d =.50) effect size which corresponds to a 3.95 change in the SDQ impairment score.

**2.12 What is the planned recruitment rate?**

At the start of the school year, the schools will send study participation consent forms to the parents and

assent forms to students. Based on the results of previous studies conducted by our team, we are expecting an overall participation rate of around 80% (20% refusing to take part).

**2.13 Are there likely to be any problems with compliance?**

In previous studies, non-compliance rates (failure to attend sessions) were so low that we didn’t need to

control for absenteeism, which we had initially planned to do to calculate an eventual dose/effect relation. The students in special classes enjoyed the pilot project workshops so much that the absenteeism rates observed in these pilot projects were extremely low. Absenteeism will nevertheless be tracked systematically and its effect on the program (tutoring and theatre) controlled if needed.

**2.14 What is the likely rate of loss to follow up?**

Loss to follow up during the school year was 5% in previous studies, although they did not include a

follow-up measurement 9 months later, which is remarquably low for the field (115). We will allow for an additional moving rate of approximately 4% and an additional drop-out rate of 3% (estimated on the basis of previous studies), giving a total rate of loss to follow up of around 12% at T2.

**2.15 How many centers will be involved?**

This is not a multicentric study. The five high schools are involved in partnership with one university

hospital (McGill University Health Centre – MUHC). The involved high schools are big institutions (up to 2000 students) belonging to francophone school boards, because these are the schools designed to welcome the newly arrived immigrants and refugees to Montréal (Law 101). On the Island of Montréal, there are a total of 27 public francophone high schools (including those in non multiethnic neighborhoods). Our sample corresponds to 20% of all francophone high schools and to around 50% of

high schools in highly multiethnic neighborhoods. The participating schools are situated geographically in different parts of the Montréal Island, although all are in socio-economically deprived areas. Three of the high schools are among the poorest on the Island (Saint-Henri, Lucien-Pagé and La Dauversière) while the two others have a mixed population profile, in that they welcome children from middle class families in their gifted programs (Saint-Luc and Saint-Exupéry) but also serve extremely poor neighborhoods (for example, the Walkley area in the case of Saint-Luc).Thus, these schools are very representative of Montréal’s multiethnic high schools (see table 1 describing the socio-demographic and cultural profile of the participating schools).

**2.16 Data analysis**

The effects of theatre intervention on the primary outcome, SDQ Impairment Impact Supplement, will

be assessed by comparing the average change in SDQ of children at the end of the 12-week intervention period in children in the theatre intervention to the children in the control group. The average change in SDQ of children in the theatre group will also be compared to the average change in SDQ for children in the tutorship group. These analyses will be performed using a linear mixed effects model (also called a hierarchical model), which will appropriately account for the within-classroom correlation of children. In addition to a term for the intervention group, the linear mixed effects model will also adjust for child age and gender.

Analyses of secondary outcomes will also be carried out using linear mixed effects models; these outcomes are: (a) change in impairment, self-rated at T2 (b) change in SDQ Global Scores, self-rated (c) change in SDQ Global Scores, teacher-rated (d) change in impairment, teacher rated (e) change in Adolescent Friendship Inventory (AFI) (f) change in School Performance. Analysis of the group x time interaction will be performed with the repeated measures (T0, T1, T2). All analyses will be conducted using the **R** statistical programming language, using the lme procedure to fit the linear mixed effects models.

**2.17 What is the proposed frequency of analyses?**

Analyses will be done after each measurement time and then overall at the end of the data collection –

that is, analyses will be performed post-intervention and after the 9-month follow-up.

**2.18 Sub group analyses**

Analyses to determine the effect of gender and age will be conducted. In the pilot study, however, there

were no differential effects of treatment in the pilot study based on sex (p = .95) or age (p = .96). Thus, although we will control for a potential effect, we do not expect it to be significant. If classes and schools differ significantly on measured socio-demographic variables describing socio-economic and cultural variables we will include them as covariates in the outcome analysis to control for variation between groups/schools.

**2.19 Pilot studies:** The first and larger pilot study was done on regular and welcoming classes. No quantitative data was obtained in the subsequent piloting of the project in special class (90). Other pilot was recently realized (at the request of Québec Ministry of education) in special classes of under- schooled youth (n = 55). In these classes, the majority of youth have suffered high exposure to trauma and separation. This pilot results confirmed that self-report impairment decreased significantly with the intervention and that the intervention appeared to be more beneficial when exposure to premigratory hardship is high (89).

**2.20 Estimated cost and duration of the trial**

The estimated cost of the proposed randomized trial is 573,819$ spread over 3 years, which is the

planned duration of the study (one year of field work; pre- and post-trial, T0 and T1), another year for

the follow-up (T2) and analyses, and a third year for finalizing the analysis, the transfer of knowledge and dissemination). This estimated cost is detailed in the budget justification.

**3. DETAILS OF TRIAL TEAM**

**3.1 Trial management**

The coordination of the trial fieldwork will be done by Sarah Fraser, Post Doctorate Fellow in

transcultural psychiatry with experience in research coordination and quantitative analysis. She will be in charge of the relations with the schools (scheduling of interventions and date collection) in collaboration with Marie-France Gauthier (Drama Therapist) and Anousheh Machouf (Psychologist) who will coordinate the drama workshops and an education PhD student who will coordinate the tutorship intervention under the supervision of Dr Petrakos. Sarah Fraser will be assisted by 2 Research Assistants (Tonje Perrson, PhD Student and another student to be determined). Data entry will be completed denominalized. Denominalized Questionnaires (T0-T1-T2) will be kept in locked facilities at the CSSS de la Montagne, accessible only to the research team. Report cards, which cannot be denominalized, will be kept in a locked file and destroyed 5 years after the completion of the study.

**3.2 Role of applicant and co-applicants**

The research team is made up of people from three universities: McGill University, Concordia

University and Sherbrooke University. Cécile Rousseau, a Clinical Researcher who specializes in medical anthropology and child psychiatry (McGill), will act as the lead researcher. She will coordinate the design of the research project and will manage the research team and relations with the partner schools. She will also be in charge of interpreting the data and writing up material for publication. Maryse Benoit is Assistant Professor in Psychology (Sherbrooke University) she has been involved for several years now in the evaluation of the theatre therapy program and is currently collaborating on a cross-country comparison of school programs for refugee children. She will be in charge of training the interviewers, will supervise the work of the research assistants and students attached to the project, and will be involved in the data analysis. Brett Thombs holds a Ph.D. in psychology and is Associate Professor in the Psychiatry Department of McGill University. He specializes in psychometrics with an expertise in psychosocial measures. He will be in charge of the analysis. Lily Hechtman, Professor of Psychiatry at McGill and a specialist in attention disorders and hyperactivity, will be the team’s clinical randomized trial specialist. She has extensive experience in this area and her expertise should be particularly useful with respect to children who have learning and behavioural problems (Design and interpretation of results). Helen-Maria Vasiliadis, MSc. Ph.D, Assistant Professor, Department of Community Health Sciences (Sherbrooke University) is an epidemiologist with training in health economics and mental health services research. She has worked with multidisciplinary teams and has had hands-on experience in evaluating the cost and effectiveness of programs. She has worked with large administrative and survey data for mental health services research purposes. She will work on the economic evaluation. Hariclia Petrakos is an Associate Professor in the department of education (Concordia University). She will coordinate the tutorship intervention and will participate in the interpretation of results. At the High school level, Hélène Grenier, a Psychoeducator at St. Luc, Éric Dion, Assistant Principal and a long-standing project collaborator and Frédérique Normand, Psychologist at Antoine-de-Saint-Exupéry will sit on the steering committee and represent the schools.

**3.3 Steering Committee of the trial:** This Committee will include 1 school-staff representative of each of the participating schools and 3 representatives of community (organisations and representative from school boards and Quebec department of education in order to discuss any emerging problems.
